# A Multiplex PCR-Based Assay for Authentication of Six Commercially Important Cephalopod Species

**DOI:** 10.3390/foods15122098

**Published:** 2026-06-11

**Authors:** Kang-Rae Kim, Hye-Jin Kim, Su Jin Park, In-Chul Bang

**Affiliations:** 1 Southeast Sea Fisheries Research Institute, National Institute of Fisheries Science, Namhae 52440, Republic of Korea; krkim89@korea.kr (K.-R.K.); ssujin@korea.kr (S.J.P.); 2 Department of Life Science, Soonchunhyang University, Asan 31538, Republic of Korea; n_shiho@naver.com

**Keywords:** cephalopod, multiplex PCR, COI, species identification

## Abstract

Cephalopod products are widely distributed as frozen raw materials or cut portions, making morphology-based species identification difficult during commercial handling and inspection. In this study, we developed a conventional multiplex PCR assay for the simultaneous identification of six commercially important cephalopod species, *Octopus vulgaris*, *O. ocellatus*, *O. minor*, *Enteroctopus dofleini*, *Dosidicus gigas*, and *Todarodes pacificus*. Species-specific forward primers and a shared reverse primer were designed from the mitochondrial cytochrome c oxidase subunit I (COI) region to generate distinct diagnostic amplicons within a single reaction. The assay successfully produced species-resolved bands of 459, 365, 248, 194, 141, and 82 bp for *O. vulgaris*, *E. dofleini*, *O. ocellatus*, *O. minor*, *D. gigas*, and *T. pacificus*, respectively, with no ambiguous overlap among diagnostic fragments. Clear and reproducible amplification was obtained at annealing temperatures of 51–54 °C, with 52 °C selected as the standard condition, indicating useful operational tolerance for routine application. The assay also retained consistent diagnostic performance down to 1 ng of template DNA per reaction. These results demonstrate that the developed multiplex PCR assay provides a simple, rapid, and gel-based method for the preliminary identification of selected cephalopod species in frozen commercial materials and may be useful for seafood inspection and market surveillance.

## 1. Introduction

Cephalopods, particularly octopus and squid, represent a high-value segment of the global seafood trade and are widely consumed across Asian and Mediterranean markets [1,2]. Squids account for the largest share of cephalopod fishery production worldwide, and commercially prominent resources include *Todarodes pacificus* in the Northwest Pacific and *Dosidicus gigas* in the Eastern Pacific [3,4]. Among octopuses, *Octopus vulgaris* is regarded as a premium seafood commodity in international markets, whereas *Octopus minor* and *Octopus ocellatus* are highly preferred edible octopuses in East Asia [5]. *Enteroctopus dofleini* is likewise an important commercial species in the North Pacific [6]. In terms of distribution, *O. vulgaris* occurs broadly in temperate to subtropical coastal waters, *O. minor* and *O. ocellatus* are centered in Korean, Chinese, and Japanese coastal systems, *E. dofleini* inhabits the North Pacific, *T. pacificus* is concentrated in the Northwest Pacific, and *D. gigas* extends across the Eastern Pacific from the California Current to Peruvian and Chilean waters [4,7].

The six species examined here commonly occur in commercial seafood channels as frozen raw materials or as portions intended for subsequent processing [8,9,10]. In commercial practice, cephalopods often enter processing facilities as frozen blocks, irregular frozen masses, or pre-cut body parts, rather than as whole fresh specimens [6,10]. Once cephalopod products are frozen, trimmed, or portioned, external taxonomic features become difficult to assess consistently, reducing the reliability of morphology-based identification in trade samples [9]. For this reason, mitochondrial cytochrome c oxidase subunit I (COI) provides a practical target for species authentication because it offers strong interspecific discriminatory power and broad applicability across animal taxa [10]. For routine screening, multiplex PCR based on a mitochondrial marker is advantageous because multiple target taxa can be assessed rapidly within a single assay format [6].

Species substitution remains a practical concern in cephalopod supply chains, particularly where products are distributed as frozen or cut materials with limited morphological traceability [11,12]. High-value cephalopods are especially vulnerable to this problem because economically important octopus and squid species become difficult to distinguish once they are frozen, sectioned, or otherwise stripped of their original diagnostic morphology [11,12]. In particular, frozen raw materials imported as blocks or fragmented tissues may be mislabeled either intentionally or unintentionally during distribution and processing [11,12]. Previous studies have shown that molecular approaches can reveal cephalopod mislabeling in retail markets and have documented cases in which premium cephalopod products were substituted with lower-cost squid resources such as jumbo flying squid [12,13]. Collectively, these factors indicate the need for a simple molecular tool that can support species verification in frozen cephalopod lots handled during distribution and inspection [11,14].

Previous molecular approaches for cephalopod species identification and seafood authentication have included DNA barcoding, species-specific PCR, real-time PCR assays, recombinase polymerase amplification combined with lateral flow assay, and DNA metabarcoding [9,11]. These methods have improved the reliability of cephalopod authentication, particularly when morphological characters are unavailable in frozen, cut, or processed products [9,12,14]. However, sequencing-based or instrument-dependent approaches may not always be optimal for rapid preliminary screening in routine inspection laboratories, supporting the need for a simple conventional PCR-based assay [11,14,15,16].

In this study, we selected six commercially important cephalopod species, *O. vulgaris*, *O. ocellatus*, *O. minor*, *E. dofleini*, *D. gigas*, and *T. pacificus*, and developed a conventional multiplex PCR assay for their simultaneous identification. We designed a COI-targeted primer panel capable of generating species-resolved amplicons within a single reaction and evaluated its analytical performance across different annealing temperatures and template DNA concentrations. This study establishes a primer system intended for the practical identification of selected cephalopod species in frozen commercial materials and related inspection settings. The assay was designed as a targeted screening method for the six predefined cephalopod species examined in this study, rather than as a universal identification system for all cephalopod taxa.

## 2. Materials and Methods

### 2.1. Sample Collection and Genomic DNA Isolation

Cephalopod samples comprising *Octopus vulgaris*, *O. ocellatus*, *O. minor*, *Enteroctopus dofleini*, *Dosidicus gigas*, and *Todarodes pacificus* were collected from seafood markets in South Korea in March 2021 (Table S1). Therefore, this study did not constitute an animal experiment requiring Institutional Animal Care and Use Committee review under the applicable Korean legal framework. In Korea, the Laboratory Animal Act defines an animal experiment as an experiment conducted on laboratory animals for scientific purposes, whereas the Animal Protection Act limits the scope of protected animals to live vertebrates. Accordingly, the use of commercially obtained dead specimens for laboratory analysis falls outside the scope of animal experimentation requiring ethical approval. Species determination was performed at the time of collection by Kang-Rae Kim, an author of this study and a researcher at the National Institute of Fisheries Science, using external diagnostic traits, such as general body form, relative proportions of the mantle and arms, fin morphology, and species-specific coloration and skin patterns, following published taxonomic descriptions and identification keys. Each specimen was examined and assigned to species by this researcher prior to molecular assay development.

For molecular analysis, muscle tissue was dissected from each individual and preserved under conditions suitable for DNA extraction. The tissue samples were first kept frozen at −20 °C, reflecting the condition in which cephalopod products are commonly distributed in commercial markets, and were then transferred to 1.5 mL microtubes filled with 99.9% ethanol. To prepare DNA templates, a freeze-ethanol preservation procedure was applied, in which frozen tissues were immersed in ethanol and maintained at 4 °C for 3 days prior to extraction. Before genomic DNA isolation, the tissues were rinsed with triple-distilled water (3DW). DNA was subsequently extracted using the HiGene™ Genomic DNA Prep Kit (Biofact, Daejeon, Republic of Korea) according to the manufacturer’s protocol.

### 2.2. Design of Species-Specific Primers for Six Cephalopod Species

COI sequences were retrieved from the mitochondrial genomes or mitochondrial sequence records of six target cephalopod species deposited in GenBank and used as references for primer design: *Octopus vulgaris* (NC_006353), *O. ocellatus* (NC_007896), *O. minor* (NC_015896), *Enteroctopus dofleini* (NC_056385), *Dosidicus gigas* (EU068697), and *Todarodes pacificus* (NC_006354). The reference sequences were aligned to identify species-discriminatory nucleotide sites within the COI region. Species-specific forward primer candidates were designed from variable regions, and diagnostic nucleotide differences were preferentially positioned near the 3′ end of each primer where possible to enhance target selectivity. A shared reverse primer was selected from a comparatively conserved region of the COI alignment to enable simultaneous amplification of the six target taxa in a single multiplex reaction. Candidate primers were evaluated based on primer length, GC content, melting temperature compatibility, expected amplicon size separation, and suitability for multiplex amplification. Primer candidates were also selected to be compatible with an expected annealing range of approximately 51–54 °C for conventional multiplex PCR, which was subsequently confirmed by empirical temperature optimization. The final primer panel was selected to generate non-overlapping diagnostic fragments that could be clearly resolved by standard 1.5% agarose gel electrophoresis.

### 2.3. Species-Specific Multiplex PCR Primer Set Amplification

PCR was performed in 20 μL reactions using the AccuPower® Multiplex PCR Pre-mix (Bioneer Inc., Daejeon, Republic of Korea) on a Bio-Rad thermal cycler (Bio-Rad Laboratories, Hercules, CA, USA). Each reaction contained 10 ng of template DNA, six species-specific forward primers, and one shared reverse primer (10 μM stock concentration for each primer). Thermal cycling consisted of 94 °C for 5 min; 34 cycles of 94 °C for 30 s, 55 °C for 30 s, and 72 °C for 30 s; followed by a final extension at 72 °C for 7 min. PCR amplification products were confirmed on a 1.5% agarose gel.

### 2.4. Determining the Efficient Annealing Temperature of a Multiplex PCR Primer Set

Multiplex PCR was performed as described in the Materials and Methods (Section 2.3), except that to identify the optimal annealing range, the annealing temperature was tested at eight settings (50, 51, 52, 54, 56, 58, 60, and 61 °C).

### 2.5. Assessment of Multiplex PCR Performance at Varying DNA Concentrations

Multiplex PCR was performed with genomic DNA from each of the six species at four template inputs (50, 10, 1, and 0.1 ng per reaction) to evaluate assay sensitivity. All other reaction conditions were identical to those described in Section 2.3. Post-PCR band intensities for each species and template DNA input were compared by digital image analysis using ImageJ software (version 1.53a). ImageJ analysis was only used to evaluate the relative intensity of amplified PCR products on agarose gel images and was not used to determine the genomic DNA concentration of the template samples.

## 3. Results and Discussion

### 3.1. Selection of COI-Based Species-Discriminatory Primer Sites

**Figure 1:**
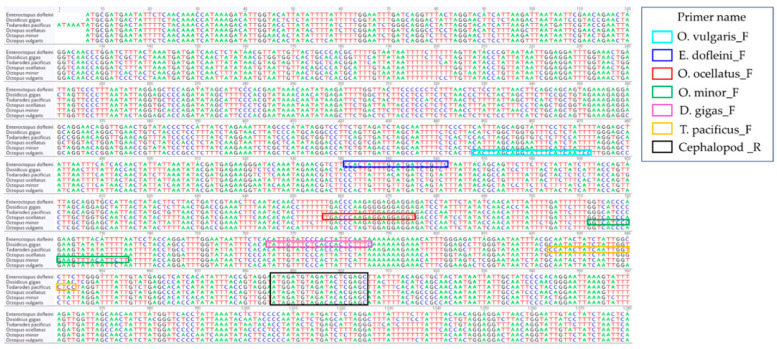
Alignment-guided design of the COI-targeted multiplex primer panel for six cephalopod species. Colored boxes indicate the binding positions of the species-specific forward primers for *O. vulgaris*, *E. dofleini*, *O. ocellatus*, *O. minor*, *D. gigas*, and *T. pacificus*, respectively. The shared reverse primer region is shown within the most conserved segment of the alignment.

Alignment of the COI region across the six target cephalopods revealed multiple nucleotide positions suitable for species-level primer discrimination. Forward primers were therefore placed in variable regions so that each target taxon carried diagnostic sequence information near the 3′ end of the primer (Figure 1), a configuration expected to suppress extension from non-target templates and thereby improve analytical specificity [17]. To support multiplex compatibility, the reverse primer was designed within a relatively conserved segment shared across the six taxa, and degenerate bases were introduced only where necessary to accommodate limited interspecific variation [18,19,20]. This primer arrangement allowed the assay to retain species selectivity while generating band sizes that could be readily distinguished on standard agarose gels [19,20].

### 3.2. Species Resolution by the Multiplex PCR Assay

**Figure 2:**
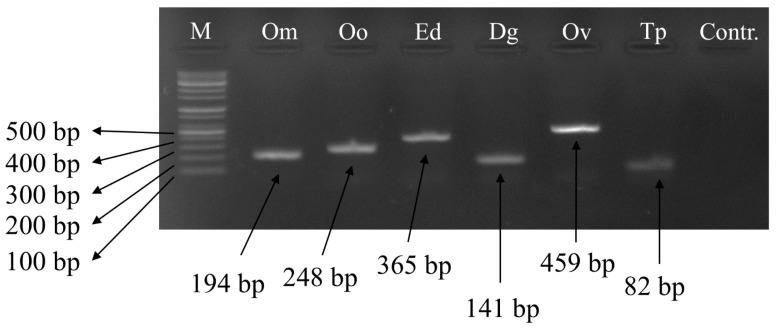
Representative multiplex PCR profiles obtained from the six target cephalopod species. M, DNA size marker; Ov, *Octopus vulgaris*; Ed, *Enteroctopus dofleini*; Oo, *Octopus ocellatus*; Om, *Octopus minor*; Dg, *Dosidicus gigas*; Tp, *Todarodes pacificus*; Contr., negative control.

The finalized primer set generated a distinct amplification product for each of the six target species in a single multiplex reaction. All amplicons were resolved at their expected sizes, namely 459 bp for *O. vulgaris*, 365 bp for *E. dofleini*, 248 bp for *O. ocellatus*, 194 bp for *O. minor*, 141 bp for *D. gigas*, and 82 bp for *T. pacificus* (Table 1, Figure 2). No ambiguous overlap among diagnostic bands was observed under the tested conditions, indicating that the size structure of the assay was appropriate for straightforward gel-based identification. These findings show that the primer panel can distinguish the six target cephalopod species under conditions where external morphological diagnosis is not feasible. Because the assay was evaluated only using the six target species, its diagnostic interpretation should be restricted to these species and to the experimental conditions tested in this study.

The utility of this assay extends beyond visual band separation because it addresses a set of cephalopod species that may be difficult to distinguish in frozen trade materials. For routine inspection work, methods are most useful when they combine reliable species resolution with low operational burden and uncomplicated result interpretation [11]. In this respect, the clear size separation among the six diagnostic amplicons is an important advantage of the assay, because it enables direct visual interpretation on standard agarose gels. Accordingly, the assay may be useful as a first-line screening tool in laboratories that process many samples and cannot rely on sequencing for every case [11,15]. Because this method is based on multiplex amplification and gel-based size discrimination, it should be interpreted as a preliminary screening tool rather than as a replacement for sequence-based confirmation. Multiplex PCR can be affected by primer interactions, differential amplification efficiency, and preferential amplification among targets, which may complicate downstream interpretation when multiple primer pairs are included in a single reaction [21,22]. Therefore, future studies should validate representative diagnostic bands by singleplex PCR or standard COI barcoding followed by Sanger sequencing, particularly for ambiguous samples, legally sensitive applications, or broader regulatory use [9]. Compared with DNA barcoding and DNA metabarcoding, the present multiplex PCR assay does not require sequencing or post-sequencing bioinformatic analysis, although it is limited to the six predefined target species [11,12]. Real-time PCR and RPA-LFA assays may provide higher analytical sensitivity or field-deployable detection, but they require specialized instruments, assay formats, or target-specific validation [11,15,23]. Therefore, the practical value of the present assay lies in its low-cost, gel-based format and its ability to simultaneously discriminate six commercially important cephalopod species in a single conventional PCR reaction. Because the assay was developed for visible cephalopod tissue materials, such as frozen blocks or cut portions, its primary purpose was rapid species-level screening of predefined target species rather than trace-level detection, quantitative analysis, or real-time PCR-based confirmation.

### 3.3. Evaluation of Annealing Temperature Conditions for the Multiplex PCR Assay

**Figure 3:**
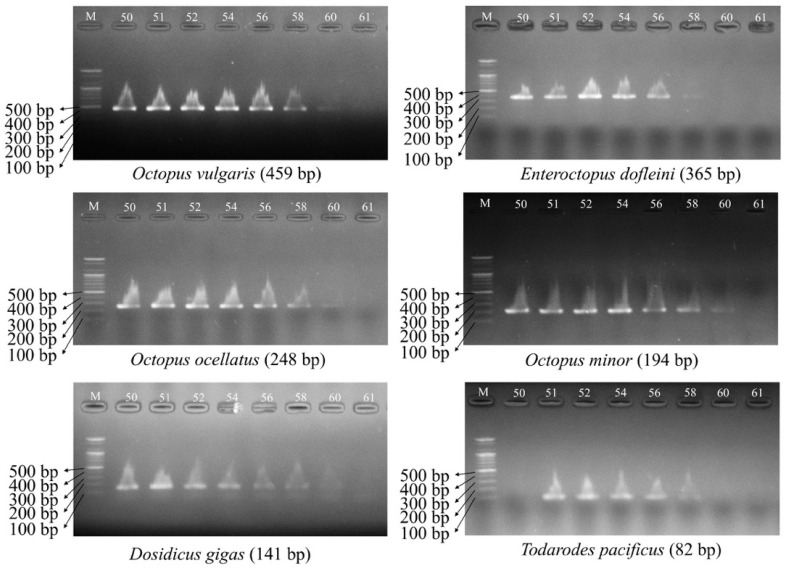
Multiplex PCR amplification patterns of the six-species cephalopod primer panel across annealing temperatures from 50 to 61 °C. M, DNA size marker; numerals above lanes indicate the annealing temperature used in each reaction. Diagnostic interpretation was based on the presence of bands at the expected species-specific amplicon sizes.

The multiplex assay was evaluated across annealing temperatures from 50 to 61 °C in order to define conditions suitable for routine application (Figure 3). Among the tested annealing temperatures, 51, 52, and 54 °C yielded the clearest and most reproducible species-diagnostic bands and were therefore selected as the optimal annealing conditions. This temperature tolerance is advantageous for practical laboratory use because it reduces sensitivity to minor run-to-run variation and facilitates method transfer between laboratories [24,25]. Accordingly, 52 °C was used as the standard annealing temperature for subsequent analyses, while 51–54 °C was considered a practical working range.

The observed tolerance across several annealing temperatures is also meaningful for practical assay deployment. In routine diagnostic or food control laboratories, minor variation in thermocycler performance, reagent lots, and sample quality can influence amplification outcomes [24]. Methods that depend on a highly restricted thermal condition may be less convenient to reproduce across laboratories, whereas assays that remain functional over a modest temperature interval are more adaptable in routine use [13,25]. The ability of the present primer panel to yield clear diagnostic bands at 51–54 °C therefore suggests that the method has useful operational tolerance for routine implementation rather than being dependent on a highly restrictive thermal condition. Although minor peak-like or curved band shapes were observed in some lanes, these features were not interpreted as additional PCR products because the diagnostic bands were detected at the expected species-specific amplicon sizes. Such minor band-shape distortion may arise from gel loading, well geometry, sample distribution, electrophoretic migration, or image acquisition conditions, which are known to affect band morphology in agarose gel electrophoresis [26].

### 3.4. Effect of Template DNA Concentration on Multiplex PCR Amplification Efficiency

**Figure 4:**
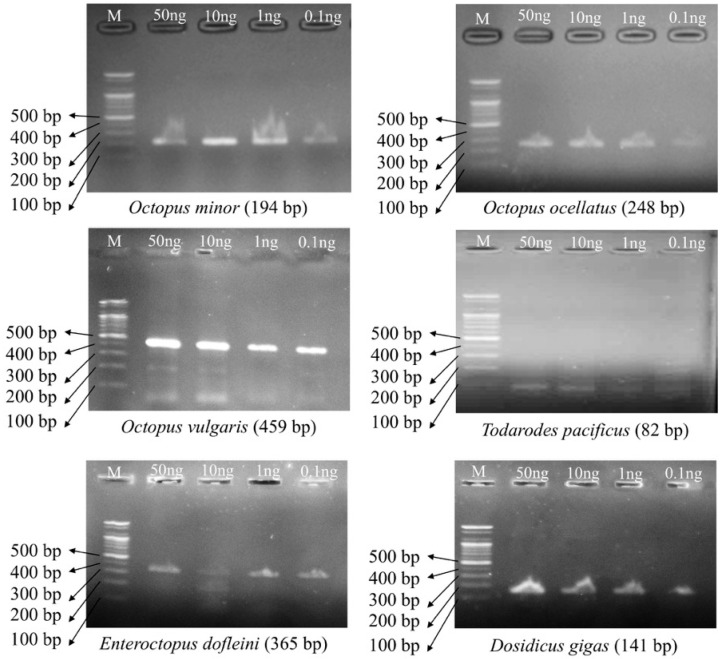
Multiplex PCR sensitivity of the six-species cephalopod assay across template DNA inputs of 50, 10, 1, and 0.1 ng per reaction. Species-specific banding patterns were evaluated to determine the lower detectable template input and amplification stability across the tested concentration series. Diagnostic interpretation was based on the presence of bands at the expected species-specific amplicon sizes rather than on minor differences in band intensity.

Species-specific amplification was observed across the tested concentration series, and clear diagnostic bands were reproducibly retained down to 1 ng of template DNA per reaction. Although amplification was still detectable at lower inputs in some cases, 1 ng was the lowest concentration that consistently produced clear diagnostic bands across species. Although band intensity generally declined as DNA input decreased, the expected diagnostic fragments remained detectable at 1 ng, which supports the sensitivity of the method for applications involving low-yield extracts or partially degraded material. Because the present assay is a qualitative endpoint multiplex PCR assay, agarose gel band intensity was not used as a quantitative measure of the initial template DNA concentration. In endpoint PCR, final product yield may be influenced by plateau-phase amplification, primer depletion, primer-pair efficiency, primer characteristics, and reaction competition rather than by template input alone [27]. Therefore, occasional differences in band brightness across the DNA concentration series, including stronger bands at lower template inputs in some lanes, were not interpreted quantitatively. The relatively faint band observed for *T. pacificus* in Figure 4 was retained as a positive diagnostic signal because it appeared at the expected amplicon size of 82 bp. Accordingly, diagnostic interpretation was based on the presence of species-specific bands at the expected sizes, rather than on absolute band intensity. Taken together, these findings indicate that the assay provides sufficient analytical sensitivity for routine species verification of commercial cephalopod products, particularly when template DNA is available at 1 ng per reaction or higher.

The consistent detection observed at 1 ng of template DNA suggests that the assay can accommodate DNA amounts likely to be encountered in commercial sample testing [15,28]. In seafood authentication, DNA quantity and quality are not always optimal, especially when tissues are frozen for extended periods or sampled from fragmented raw materials [28,29]. Under such conditions, an assay does not necessarily need to detect extremely low copy numbers if it can reliably produce interpretable results from typical extracted DNA concentrations encountered in routine monitoring [15]. In practical terms, this result suggests that the assay should remain usable for many frozen-material samples, including those yielding less DNA than freshly collected tissue.

This study should also be interpreted in light of several limitations. This assay was assessed using authenticated reference materials and controlled DNA inputs, whereas its performance in highly processed commercial products was not examined. Since processing can compromise DNA quality, further validation is required for broader application to processed samples [30,31]. Moreover, expanded cross-reactivity testing against closely related and commercially relevant non-target cephalopod species would further support analytical specificity and would be necessary before applying the assay to broader regulatory testing or expanded market-surveillance programs [14,28,32]. Further evaluation using more diverse commercial products will be necessary to define the scope of this method in routine food testing [28,32]. Future validation should include repeatability, reproducibility, mixed-sample testing, broader non-target testing, processed-product evaluation, and inter-laboratory assessment before regulatory implementation.

## 4. Conclusions

We developed a conventional COI-targeted multiplex PCR assay capable of simultaneously identifying six commercially important cephalopod species, *O. vulgaris*, *O. ocellatus*, *O. minor*, *E. dofleini*, *D. gigas*, and *T. pacificus*, in a single reaction. The assay generated clearly separated diagnostic amplicons, remained stable across the tested annealing conditions, and consistently detected target DNA at template inputs of 1 ng or higher. Using species-directed forward primers together with a common reverse primer in the COI region, the assay offers a simple gel-based option for identifying frozen cephalopod materials in routine testing contexts. Its applicability to processed seafood products remains to be further validated. This primer set may serve as a practical preliminary screening option for laboratories involved in seafood inspection, especially when the rapid verification of frozen materials corresponding to the six target species is required.

## Figures and Tables

**Table 1:** COI-targeted species-specific primers used in the multiplex PCR assay for six commercially important cephalopods.

Primer Name	Scientific Name	Sequence (5′-3′)	Primer Direction	Expected Amplification Product Size (bp)
O. vulgaris_F	*Octopus vulgaris*	CACTTAGCAGGTATTTCATCAATCC	Forward	459
E. dofleini_F	*Enteroctopus dofleini*	CCACTATTTGTATGATCTGTTC	Forward	365
O. ocellatus_F	*Octopus ocellatus*	TGACCCAAGAGGAGGAGGT	Forward	248
O. minor_F	*Octopus minor*	GGTCATCCCGAAGTTTACATTCTT	Forward	194
D. gigas_F	*Dosidicus gigas*	TATTGTTTCTCACCACTCTTTC	Forward	141
T. pacificus_F	*Todarodes pacificus*	CCATACTATCAATTGGTCTCC	Forward	82
Cephalopod_R	-	GCWCGWGTRTCWACATCYAT	Reverse	-

## Data Availability

The data presented in this study are available on request from the corresponding author.

## Supplementary Materials

The following supporting information can be downloaded at: https://www.mdpi.com/article/10.3390/foods15122098/s1, Figure S1, Quantitative band measurements for six cephalopod species.; Table S1, Sampling sites of six cephalopod species in the study.
