# Supplementary material for: A Multiplex PCR-Based Assay for Authentication of Six Commercially Important Cephalopod Species
Source: Foods. 2026 Jun 11;15(12):2098. doi: 10.3390/foods15122098 (PMC13297775; doi:10.3390/foods15122098)
Supplement: Supplementary file 1 [file foods-15-02098-s001.zip › foods-4285601-supplementary.pdf]

Table S1. Sampling sites of six cephalopod species in the study

Scientific name	Location	Location	Sample date
Octopus ocellatus	Incheon	37°28'12″ N, 126°35'03" E	2021.03
Octopus minor	Mokpo	34°46'43″ N, 126°22'59" E	2021.03
Enteroctopus dofleini	Gangneung	37°47'45″ N, 129°02'48" E	2021.03
Octopus vulgaris	Namhae	34°42'11″ N, 128°02'50" E	2021.03
Todarodes pacificus	Sokcho	38°12'40″ N, 128°36'03" E	2021.03
Dosidicus gigas	Republic of Peru	5°05'00″ S, 81°06'01" W	2021.03

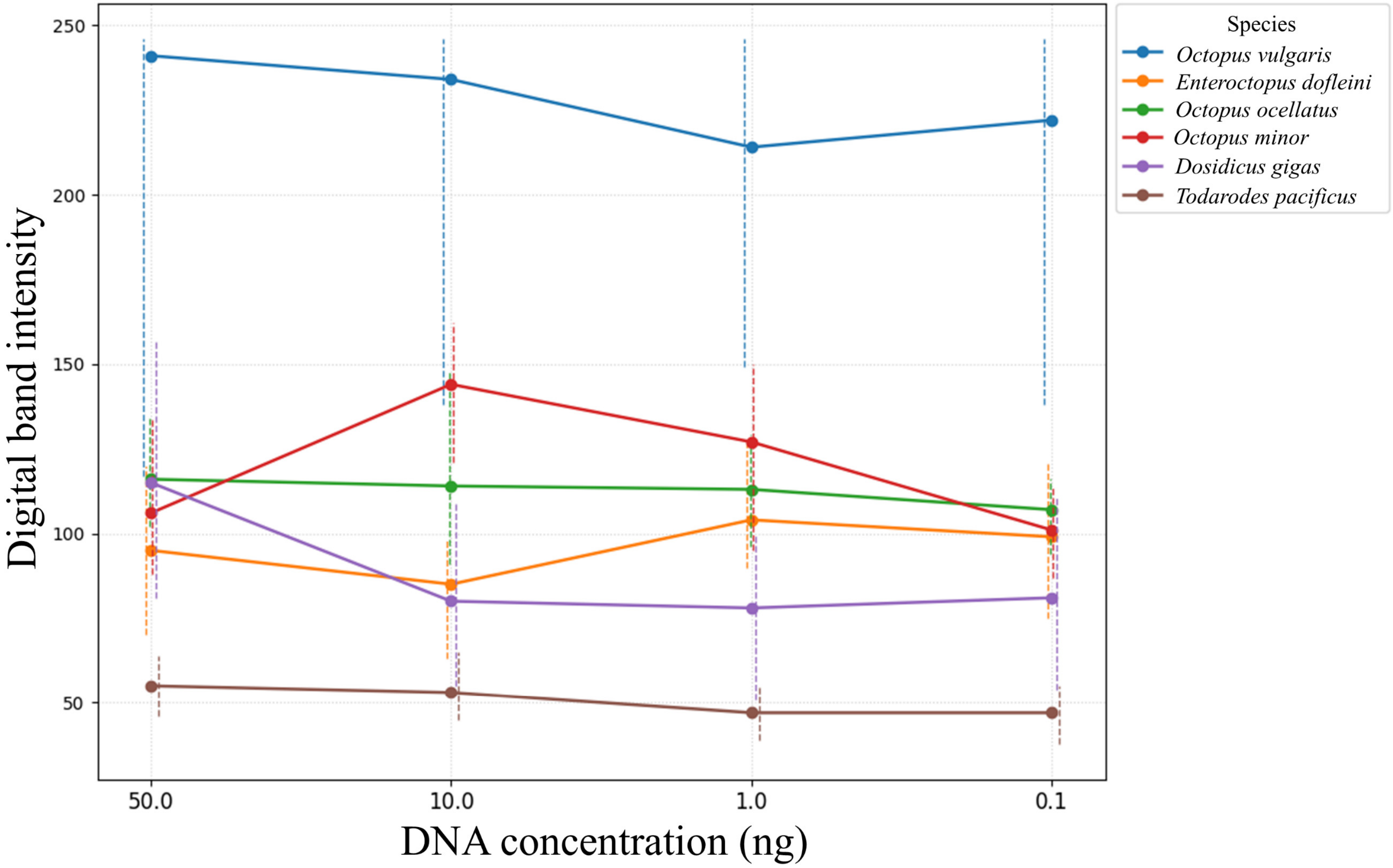

Figure S1. Quantitative band measurements for six cephalopod species. Line colors denote the species-specific digital quantification values. The error bars show the minimum and maximum ranges, while the central line marks the average.
